# Family and home influences on children’s after-school and weekend physical activity

**DOI:** 10.1093/eurpub/cks160

**Published:** 2012-11-19

**Authors:** Alison M. McMinn, Simon J. Griffin, Andrew P. Jones, Esther M. F. van Sluijs

**Affiliations:** 1 Medical Research Council Epidemiology Unit, Cambridge, UK; 2 UKCRC Centre for Diet and Activity Research, Institute of Public Health, Cambridge, UK; 3 School of Environmental Science, University of East Anglia, Norwich, UK

## Abstract

**Background:** Family- and home-related factors have been shown to be associated with children’s physical activity (PA), but may be time-dependent. Here we investigate whether family- and home-related correlates of children’s PA are different for the after-school period on weekdays than for the weekend. **Methods:** Data on 21 family- and home-related variables and objectively measured PA (Actigraph GT1M) were available from 1608 Year 5 children (9–10 years old) from 92 schools in Norfolk participating in the SPEEDY (Sport, Physical activity and Eating behaviour: Environmental Determinants in Young people) study. Multi-level multiple linear regression was used to quantify cross-sectional associations between the family/home variables and average min per day of moderate-to-vigorous PA (MVPA, ≥2000 counts/min) after school on weekdays and at the weekend. Models were additionally adjusted for age, sex, BMI *z*-score and registered accelerometer wear time. **Results:** After-school MVPA was associated with parent education (ß: −1.1; 95% CI −2.0 to −0.2), being allowed to play out in the neighbourhood (ß: 1.3; 0.7–1.8), restrictions on walking/cycling to friends’ houses (ß: −1.1; −1.6 to −0.7), restrictions on sedentary behaviour (ß: −0.3; −0.5 to −0.02) and family social support (ß: 1.0; 0.7–1.3). Weekend MVPA was associated with number of siblings (ß: 2.6; 0.5–4.8), family encouragement (ß: 1.1; 0.2–2.0) and family social support (ß: 1.5; 0.5–2.5). **Conclusion:** Family social support is positively associated with children’s out-of-school PA both at weekdays and in weekends. However, rules and restrictions appear to be important only on weekdays. The results of this study merit consideration when identifying appropriate timing of PA-promotion strategies.

## Introduction

Physical activity in childhood and adolescence has been associated with a range of health benefits including the prevention of obesity, improvements in bone mineral density, a reduction in metabolic and cardiovascular disease risk factors and positive effects on mental health.^1–5^ Promoting physical activity is therefore a key focus of public health policy in developed countries.^6^^,^^7^ To aid intervention development, it is important to understand the factors that influence children’s physical activity. The family and home environment may affect children’s physical activity in a number of ways. Parents can act as role models, sources of support and gatekeepers through their control over children’s opportunities for physical activity.^8^ Similarly, other family members are also likely to influence children’s physical activity through role modelling and social support. The home environment can additionally impact on physical activity behaviour through the provision of opportunities to be physically active (e.g. having play equipment available) or to be sedentary (e.g. having a television, computer and other electronic entertainment devices).

Previous findings on the associations between various factors in the family and home environment and children’s physical activity have been somewhat mixed,^9–11^ with indications that results differ by child’s age, sex and ethnicity.^12^Variations in the measurement method and validity of the physical activity, family and home environment variables used are also likely to contribute to the inconsistency in results.^12^ An additional contributing factor might be that family- and home-related correlates of children’s physical activity are time- and context-specific. In this case, associations may be expected to differ between studies depending on exactly which aspect of physical activity behaviour has been measured.

There has been some work to explore whether family- and home-related correlates of children’s physical activity differ according to the setting or time of day that the activity occurs. For example, Ommundsen *et al.*^13^ investigated associations between several psychological and social variables and children’s and adolescents’ self-reported commuting mode to school, leisure time physical activity and informal game playing at school. Although some psychological variables were consistently associated with the different types of activity, parental support and parental licence for children to go out were both positively associated with leisure time physical activity but not with informal game playing at school nor commuting mode.^13^ Heitzler *et al.*^14^ and Spinks *et al.*^15^ investigated associations with organized vs. free-time physical activity and structured vs. unstructured physical activity, respectively. Both reported that parental support was more often associated with organized or structured physical activity. A limitation of these studies, however, is the use of self-reported measures of physical activity, which are known to be less precise than objective measures,^16^ and could be correlated with the exposure variables. The use of independent measures of exposure and outcome is therefore encouraged.^12^

Objective measures cannot distinguish between physical activity behaviours as such but can be used to investigate associations with physical activity at different times of the day or week. Carver *et al.*^17^ investigated associations between parental restrictions on outdoor physical activity and 10–11-year-old children’s objectively measured weekday (outside of school hours) and weekend physical activity. For girls, they reported associations between restrictions and weekday, but not weekend, physical activity, whereas for boys, the reverse was true although there were also fewer associations in boys.^18^ Conversely, when Page *et al.*^19^ investigated associations between independent mobility (both locally and in the wider area) and objectively measured weekday and weekend physical activity in a sample of UK children, they reported associations only with weekday physical activity for boys but for both weekday and weekend physical activity for girls. This limited evidence shows that a more detailed investigation into this question using a wider range of potential correlates is warranted.

Investigating time-specific correlates of physical activity is important, as evidence suggests that declines in physical activity during late childhood are not evenly spread across the day and week, with larger decreases observed on weekend days and during leisure time on weekdays.^20^ We therefore set out to investigate the family and home environment correlates of children’s objectively measured physical activity after school on weekdays and at the weekend.

## Methods

### Study design and participants

The SPEEDY study (Sport, Physical activity and Eating behaviour: Environmental Determinants in Young people) is a population-based study, investigating individual and collective factors associated with physical activity and dietary behaviour in schoolchildren in the county of Norfolk, England, UK. Ethical approval for this study was obtained from the University of East Anglia Faculty of Health Ethics Committee. The cross-sectional analyses presented here use data collected between April and July 2007 when the children were in Year 5 (9–10 years old).

Full details on participant recruitment, study procedures and sample representativeness for the SPEEDY study have been described elsewhere.^21^ Briefly, schools in Norfolk were purposively sampled to achieve heterogeneity in urban and rural locations. From 227 schools that were eligible for recruitment, 157 were approached and measurement sessions were conducted at 92 schools. All Year 5 children (*n* = 3619) at the 92 schools were invited to participate. In total, parents of 2064 children provided written informed consent for participation (57% response rate).

### Data collection

A team of trained staff visited schools to take physical measurements, administer two child questionnaires, fit accelerometers and distribute a home pack (containing a parent questionnaire and food diary) to each child. Participants were asked to return the completed home packs and accelerometers to school 8 days after the visit.

Standardized protocols were used to collect data on children’s height and weight. Height was measured to the nearest millimetre using portable Leicester height measures, weight to the nearest 0.1 kg using a non-segmental bio impedance scale (Tanita, type TBF-300A). Body mass index (kg/m^2^) was calculated and children’s weight status was derived using established protocols.^22^ Age and gender were self-reported during the measurement session, and ethnicity was parent-reported using the UK standard classification.

#### Physical activity

Physical activity was measured using waist-mounted ActiGraph accelerometers (GT1M, ActiGraph LCC, Pensacola, FL, USA). Accelerometry has previously been validated for use in children.^23^^,^^24^ Children were fitted with the monitor during the measurement session and instructed to wear it continuously on their right hip throughout waking hours for 7 consecutive days, except when participating in water-based activities. The accelerometer was set to record activity counts in 5-s epochs. Data were cleaned using specially designed software (MAHUFFE Processing Software, available at: http://www.mrc-epid.cam.ac.uk/Research/Programmes/Programme_5/index.html) to remove the first day of data collection and any days with <500 min of recording (defined as a valid day).^25^ Periods of ≥10 min that had continuous zero counts were coded as non-wear time, and data recorded after 11 pm and before 6 am were also excluded to focus on day time activity. Participants with <3 valid days (including a weekend day) were excluded; this cut-off was based on previous research in British schoolchildren.^25^

The two outcome variables for this analysis were average min per day of moderate-to-vigorous physical activity (MVPA) after school (3–11 pm) on weekdays (after-school MVPA) and average min per day of MVPA at the weekend (weekend MVPA). Minutes of MVPA were defined as min with ≥2000 counts, corresponding to a walking pace of about 4 km/h in children.^26^ This outcome measure was chosen to link closely with the current physical activity guidelines for children, which focus on engaging in activity of at least moderate intensity.^6^

#### Family and home environment variables

A total of 21 family and home environment variables were included in this analysis, based on previously used questions where possible (see table 1). Factors with limited variation (defined as >90% of responses in one category or direction) were excluded from analyses. These included ethnicity (96.2% white), whether children always had to tell their parents where they were going (93.3% yes), the frequency that parents allowed their child to play out after dark (91.3% never/rarely) and whether or not the child had a garden, television or computer at home (98.7, 99.5 and 96.2% yes, respectively).

**Table 1:** Description of family and home environment variables

Variable name^a^	Description and/or coding
Socio-demographics (parent-reported)	
Parent education	Collected in 14 categories then coded as: low, medium, high.
House ownership	Coded as: own home, rent home.
Parents at home	Number of parents reported living at home; coded as: one, two.
Siblings	Number of siblings reported living at home; coded as: one, two, three or more.
Maternal characteristics and behaviour (parent-reported)	
Mothers’ age	In years.
Mothers’ BMI	Calculated from height and weight (kg/m^2^).
Mothers’ PA^27^	Previously validated index^27^ based on leisure time PA and PA at work. Coded as: inactive, moderately inactive, moderately active, active.
Mothers’ time in sedentary activities^28^	Sum of responses (none to 4+ h a day) to four questions on time spent watching TV and using computer outside of work h. Score range: 4–24.
Parental attitudes, rules and restrictions (parent-reported)	
Allowed to play anywhere in neighbourhood	Frequency parents allow child to play out anywhere in neighbourhood. Score 1 (never) to 5 (very often).
Restrict child playing outside^29^	Frequency child is restricted from playing outside. Coded as: never, rarely, sometimes, often/very often (latter two categories combined and NA coded to missing due to small numbers).
Restrict child walking/cycling to friends’ houses^29^	Frequency child is restricted walking or cycling to friends’ houses. Coded as: never, rarely, sometimes, often/very often (combined due to small numbers), not applicable (NA). Variable treated as continuous (1–5) in analysis due to there being evidence of a linear association when treated as categorical.
Restrict sedentary activities^29^^,^^30^	Sum of responses [NA/never (combined), rarely, sometimes, often, very often] to three questions about frequency parents restrict their child watching TV, playing computer games and using the computer. Score range: 3–15 (α 0.82)
Responsibility for child PA	View of who should take main responsibility for children’s PA. Coded as: parents, other (school, child, other).
Indoor house rules^29^^,^^30^	Sum of responses (never to very often) to six questions regarding indoor rules (e.g. relating to meal times, bed time and playing inside). Score range: 5–30 (α 0.58)
Home social environment (child- and parent-reported)	
Family encouragement^31–33^	Sum of responses (never/hardly ever to everyday) to questions on how often family members encourage child PA, tell child PA is good for their health and that they’re doing well at PA. Child-reported. Score range: 3–12 (α 0.69).
Family social support^31^^,^^32^^,^^34^	Sum of responses (never/hardly ever to everyday) to questions on how often family members do PA with child, watch child do PA, take child to PA places. Child-reported. Score range: 3–12 (α 0.65).
Family activities^29^	Sum of responses (0–4+) to nine questions on how many times a week family members do various activities together (e.g. eat meals, watch TV, go to the park). Parent-reported. Score range: 9–27.
Home physical environment (child-reported)	
Play equipment	Play equipment in the garden; coded as yes, no.
Dog	Dog at home; coded as yes, no.
Games console^34^	Games console at home; coded as yes, no.
Electronic equipment in the bedroom^34^	Sum of responses (yes/no) to whether child has a TV, games console or desktop computer in their bedroom at home. Score range: 0–3.

NA, not applicable; PA, physical activity.

a: References are given for the sources of questions, where applicable.

### Statistical analyses

Only participants providing sufficient data for both the after-school and weekend analyses were included, and similar analyses strategies were applied for both outcomes. Characteristics of those participants included in and excluded from analyses were compared using independent *t*-tests for continuous variables and chi-squared tests for categorical variables.

Multi-level linear regression was used for all analyses to allow for the potential lack of independence of MVPA between children within the same school. Simple associations between each family- and home-related variable and MVPA (without adjustment for covariates) were first explored. Associations with a *P*-value of ≤0.05 were then selected for inclusion in a multiple multi-level linear regression model. Selected factors were entered into the model in blocks, guided by an ecological framework of health behaviour.^35^ First entered were age, sex, BMI *z*-score and relevant registered accelerometer wear time, included for their potential confounding effects. Next, socio-demographic factors were entered, followed by maternal characteristics and behaviour, then parental attitudes, rules and restrictions, then home social environment factors and, lastly, home physical environment factors. Any variable with a non-significant *P*-value was removed before adding the next block. If more than one variable within a block was non-significant, these were removed one at a time, starting with the highest *P*-value, to see if the removal of one variable changed the statistical significance of those remaining in the model.

## Results

A total of 1608 (77.9%) participants had at least 3 valid days of physical activity data including a weekend day as well as a completed parent questionnaire and formed the sample for the analyses presented here. Characteristics of these participants are displayed in table 2. No significant differences between those excluded from (*n* = 456) and included in analyses were identified for sex, age, BMI *z*-score, ethnicity or parent education level.

**Table 2:** Participant characteristics (*n*****=****1608)

Variable	% or mean ± SD
Gender (% male)	44.7
Age (years)	10.3 ± 0.3
Ethnicity (% white)	96.2
BMI *z*-score	0.4 ± 1.1
Weight status (%)	
Overweight	17.5
Obese	4.9
Parent education	
Low	38.2
Medium	41.0
High	20.8
After-school MVPA (min/day)	37.3 ± 15.5
Weekend MVPA (min/day)	74.7 ± 35.1

### Associations with after-school MVPA

In the unadjusted multi-level analyses, parental education, restrictions on walking or cycling to friends’ houses and restrictions on time spent in sedentary activities were all negatively associated with after-school MVPA (see table 3). Mother’s physical activity, being allowed to play out anywhere in the neighbourhood, indoor house rules, family encouragement, family social support, having a games console and having electronic entertainment equipment in the bedroom were all positively associated with after-school MVPA.

**Table 3:** Simple associations between family and home variables and after-school and weekend MVPA

Variable	After-school MVPA^a^	Weekend MVPA^a^
	ß (95% CI)	ß (95% CI)
Demographics		
Parent education (score: 1–3)	−0.8 (−1.3 to −0.3)**	−1.0 (−3.6 to 1.7)
House ownership (rent vs. own)	2.0 (−0.1 to 4.0)	0.3 (−3.7 to 4.4)
Parents living at home (2 vs. 1)	−0.3 (−2.4 to 1.7)	3.0 (−1.4 to 7.4)
Number of siblings (score: 0–3+)	0.1 (−0.9 to 1.1)	3.1 (0.7 to 5.6)**
Maternal characteristics and behaviour		
Mother’s age	0.0 (−0.2 to 0.1)	−0.2 (−0.5 to 0.2)
Mother’s BMI	0.0 (−0.1 to 0.2)	−0.3 (−0.7 to 0.1)
Mother’s PA (score: 1–4)	0.7 (0.0 to 1.5)*	−0.1 (−2.0 to 1.8)
Mother’s time in sedentary activities (score: 4–24)	−0.1 (−0.4 to 0.1)	−0.5 (−1.2 to 0.1)
Parental attitudes, rules and restrictions		
Allowed to play anywhere in the neighbourhood (score: 1–5)	1.9 (1.2 to 2.5)***	0.7 (−0.6 to 2.0)
Restrict child playing outside (score: 1–4)	0.0 (−1.0 to 1.0)	−1.3 (−3.3 to 0.7)
Restrict child walking/cycling to friends’ houses (score: 1–5)	−0.4 (−0.7 to −0.1)**	−1.4 (−2.6 to −0.1)*
Restrict sedentary activities (score: 3–15)	−0.5 (−0.8 to −0.2)***	−0.3 (−0.9 to 0.3)
Responsibility for child PA (‘other’ vs. parents)	1.7 (−0.7 to 4.0)	2.0 (−4.2 to 8.2)
Indoor house rules (score: 5–30)	0.3 (0.0 to 0.5)*	−0.2 (−0.8 to 0.4)
Home social environment		
Family encouragement (score: 3–12)	0.7 (0.4 to 1.1)***	2.1 (1.3 to 2.9)***
Family social support (score: 3–12)	1.3 (0.9 to 1.7)***	2.9 (2.0 to 3.8)***
Family activities (score: 9–27)	0.2 (−0.1 to 0.6)	0.8 (0.1 to 1.5)*
Home physical environment		
Garden play equipment (yes vs. no)	0.3 (−1.7 to 2.3)	2.7 (−1.8 to 7.3)
Dog at home (yes vs. no)	0.8 (−0.9 to 2.6)	2.9 (−0.4 to 6.2)
Games console (yes vs. no)	3.2 (1.0 to 5.4)**	4.7 (0.0 to 9.4)*
Electronic equipment in bedroom (score: 0–3)	1.7 (0.8 to 2.5)***	2.4 (0.4 to 4.5)*

Significant associations are shown in bold.

a: Results displayed are adjusted for clustering in schools only. Owing to missing data for individual exposure variables, numbers ranged from 1460 to 1608.

*P < 0.05, **P ≤ 0.01, ***P ≤ 0.001.

Five variables remained significantly associated with after-school MVPA in the final regression model (see table 4). Children who were allowed to play out anywhere in the neighbourhood more frequently and who received higher levels of family social support were more physically active in the after-school period, whereas those whose parents had a higher level of education and put greater restrictions on them for both walking/cycling to friends’ houses and taking part in sedentary activities were less physically active. This final model (including age, sex, BMI *z*-score and registered accelerometer wear time) explained 26.2% of the variance in after-school MVPA (the model with age, sex, BMI *z*-score and registered wear time alone explaining 20.1%).

**Table 4:** Adjusted associations between family and home variables and after-school and weekend MVPA

Variable	β (95% CI)
After-school MVPA^a^	
Parent education (score: 1–3)	−1.1 (−2.0 to 0.2)**
Allowed to play anywhere in the neighbourhood (score: 1–5)	1.3 (0.7 to 1.8)***
Restrict child walking/cycling to friends’ houses (score: 1–5)	−1.1 (−1.6 to −0.7)***
Restrict sedentary activities (score: 5–15)	−0.3 (−0.5 to −0.02)*
Family social support (score: 3–12)	1.0 (0.7 to 1.3)***
Weekend MVPA^a^	
Number of siblings (0–3+)	2.6 (0.5 to 4.8)*
Family encouragement (score: 3–12)	1.1 (0.2 to 2.0)**
Family social support (score: 3–12)	1.5 (0.5 to 2.5)**

a: Models adjusted for clustering in schools, age, sex, BMI *z*-score, average registered accelerometer wear time on weekdays after-school (3–11 pm) or on weekend days, as applicable, and all other variables for which results are displayed.

*P < 0.05, **P ≤ 0.01, ***P ≤ 0.001.

### Associations with weekend day MVPA

For weekend MVPA, positive associations were identified with number of siblings, family encouragement, family social support, family activities, and having a games console at home and electronic entertainment equipment in the bedroom in the unadjusted multi-level analyses (see table 3). Additionally, restricting children walking or cycling to friends’ houses was negatively associated with weekend MVPA. Three of these variables remained significant in the final model of the multi-level multiple regression (see table 4). Children with a greater number of siblings and higher levels of family encouragement and social support were more physically active on weekends. The final model (including age, sex, BMI *z*-score and registered accelerometer wear time) explained 17.2% of the variance in weekend MVPA (the model with age, sex, BMI *z*-score and registered wear time alone explained 15.2%).

## Discussion

This article investigated whether family- and home-related correlates of children’s physical activity differed between the after-school period and at weekends. A greater number of correlates were identified for after-school than weekend physical activity, suggesting that the family and home environmental factors investigated here may have a greater role to play in children’s physical activity during this period. Specifically, parental rules and restrictions seem to be more important at this time. There was also a negative association with parent education for after-school MVPA that was not apparent at the weekend while having siblings was associated with doing more physical activity at the weekend but not after school. Only family social support, characterized by instrumental rather than verbal support, was found to be associated with both outcomes. This reinforces previous findings.^36^^,^^37^ Overall, the findings of the current study suggest that slightly different approaches might be needed to promote physical activity at different times of the week but that family social support is worthwhile targeting in general to increase physical activity levels.

During the after-school period, but not at the weekend, children were more physically active if they were allowed to play out anywhere in the neighbourhood more and less restricted in walking or cycling to friends’ houses. During the after-school period, children who are not allowed out may replace that time with being in the home whereas at the weekend they may go elsewhere to do physical activity (e.g. for organized sports). Interestingly, parents who answered ‘not applicable’ for how often they restricted their child walking/cycling to friends’ houses had the least active children during the after-school period, even compared with those who answered ‘never’. This may reflect that parents who answered ‘not applicable’ simply do not allow their child to ever do this behaviour, therefore applying the greatest level of restriction; however, we can only speculate on this. There is a general perception that children’s freedom for playing outside and travelling independently to local places has declined over recent decades owing, at least in part, to concerns about children’s safety.^38^ Increasing opportunities for children to be active in the after-school period in a safe environment that parents are comfortable with may therefore help to increase physical activity during this time. Approaches to achieve this may vary by the type of environment children live in.^39^

The observation that the level of restriction of sedentary pastimes is negatively associated with after-school physical activity seems counterintuitive but may be a result of reverse causality. It is likely that parents of children who spend more time in sedentary pastimes may, in turn, place greater restrictions on their children than parents of children who spend less time on these pursuits. Salmon *et al.*^30^ also showed that children whose parents felt that their child’s television watching must be supervised were more likely to have low activity levels.

Parent education was significantly negatively associated with after-school physical activity but not associated with weekend physical activity. This may reflect differences in the type of activities children in different socio-economic groups participate in during the after-school period but suggests that these differences do not extend into the weekend. Evidence from systematic reviews shows that there is no consistent association between parental education and children’s activity level, with the majority of studies reporting either a positive or no correlation.^9^^,^^10^ This may reflect the fact that studies have generally investigated overall physical activity.^10^^,^^11^ Although it might not be the case that a particular socio-economic group is at higher risk of having low activity levels overall, the results from this study indicate that approaches to increase physical activity levels might need to be tailored slightly for specific socio-economic groups.

We found relatively few associations with weekend physical activity, leaving much of the variance in weekend MVPA unexplained. This may reflect that physical activity undertaken at the weekend is more likely to take place away from the home and so other factors, external to the family and home environment, may be more important for weekend physical activity. The variance explained for after-school physical activity was greater, but the variables in the final model still only explained a fairly small proportion of the variance (∼6%) on top of age, sex, BMI *z*-score and registered accelerometer wear time. This suggests that for after-school physical activity there are also other influences not included in our analyses that are important. None of the home physical environment variables considered remained significantly associated with either after-school or weekend physical activity in the multiple regression analysis. Other physical environmental factors outside of the home may therefore be more important.

This study is one of the first investigating differences in correlates of physical activity over the week. Strengths include the use of an objective measure of physical activity in a large population-based sample of children living in heterogeneous environments and the inclusion of multiple exposure variables to assess the relative importance of different correlates. We used validated and previously applied measures of our exposure variables where possible. We conducted multiple tests, which may have increased the likelihood of chance findings, but considered adjusting for multiple testing not appropriate.^40^ In addition, although accelerometers are a more valid measure of physical activity than self-reported measures, when hip-worn they are known to be limited in picking up lower limb-based activities such as cycling and cannot pick up water-based activities. The limited amount of variance explained by non-demographic factors is consistent with previous correlates studies assessing physical activity objectively,^37^^,^^41^ and has been previously highlighted in relation to parental influences.^42^ However, the use of an independent measure of exposure and outcome is important to avoid issues related to correlated error,^12^^,^^43^ and should be encouraged. In addition, this article only considered the influence of factors within one potential domain (that of the home environment), and the authors acknowledge the importance of influences from other domains as well. Other limitations include the cross-sectional design and the under-representation of boys and obese children in the SPEEDY sample,^21^ which may impact on the representativeness of these findings.

## Conclusions

Using data from the SPEEDY study, we identified that family social support is important for children’s physical activity in leisure time throughout the week, but other influences appear to vary across the week. Parental rules and restrictions appear to have a greater influence on after-school physical activity whereas, at the weekend, the family social environment seems to be more important for physical activity. Further research using longitudinal data is needed to confirm the observed associations, but those developing interventions to promote physical activity may want to consider the times at which to target the intervention and tailor the targeted correlates accordingly.
